# Annuloaortic Ectasia and Arteritis: Clinical Features, Treatments of Choice, and Causative Relations

**DOI:** 10.21470/1678-9741-2018-0252

**Published:** 2019

**Authors:** Shi-Min Yuan, Hong Lin

**Affiliations:** 1 Department of Cardiothoracic Surgery, The First Hospital of Putian, Teaching Hospital, Fujian Medical University, Putian, Fujian Province, People’s Republic of China.; 2 Department of Cardiology, The First Hospital of Putian, Teaching Hospital, Fujian Medical University, Putian, Fujian Province, People’s Republic of China.

**Keywords:** Aortic Aneurysm, Arteritis, Giant Cell Arteritis, Takayasu Arteritis

## Abstract

The roles that aortitis plays in the development of annuloaortic ectasia (AAE) remain uncertain, while clinical features of AAE in arteritis are largely unknown. This study was designed to highlight the clinical features of AAE, the treatments of choice, and the causative relations between aortitis and AAE. The morphology of the aortic valve leaflets was normal in half of the patients, while the valves were thin and overstretched in the other half. Most patients had an aortic aneurysm. Half of the patients had severe aortic valve insufficiency, and one-quarter of them had dilation of the sinuses of Valsalva. Takayasu arteritis was prone to develop coronary artery lesions, whereas giant cell arteritis were not. Aortic branch lesions in Takayasu arteritis were stenotic or occlusive in 92.9% of the patients, while in giant cell arteritis, they were all dilated lesions. Most patients (94.7%) required surgical treatment with steroid therapy. However, long-term follow-up results showed a higher anastomotic dehiscence rate, particularly in patients with Takayasu arteritis. Further morphometric and pathological research on AAE in arteritis should be undertaken, and more feasible measures should be warranted for preventing postoperative anastomotic dehiscence.

**Table:** 

Abbreviations, acronyms & symbols				
AA	= Ascending aorta		GCA	= Giant cell aortitis
AAE	= Annuloaortic ectasia		IgA	= Immunoglobulin A
AVR	= Aortic valve replacement		IgG	= Immunoglobulin G
C3	= Component 3		PRISMA	= Preferred Reporting Items for Systematic Reviews and Meta-analyses
C4	= Component 4		TA	= Takayasu arteritis
CABG	= Coronary artery bypass grafting			
DA	= Descending aorta			

## INTRODUCTION

In 1961, Ellis et al.^[1]^ proposed the concept of annuloaortic ectasia (AAE) representing an entity of dilation of the proximal ascending aorta, sinuses of Valsalva, and aortic annulus with poor coaptation of the valvular leaflets and aortic regurgitation. In the cohort of AAE, patients may have aortic dissection, aneurysm, or rupture^[2,3]^. Moreover, the echocardiographic features of AAE were marked dilation of the aortic root with a unique posterior movement (“paradoxical” motion) of the posterior aortic wall during the early-middle ejection period in 75% of the patients, and premature systolic partial closure of the aortic valve in all of the patients^[4]^. The etiological evaluations suggested that cystic medial necrosis is the most frequent etiology of AAE^[5]^, and less common etiologies may include atherosclerosis, luetic aortits, chronic aortic dissection, and syphilitic aortic aneurysm^[6]^. AAE was occasionally associated with connective tissue disorders, such as Marfan, Ehlers-Danlos^[7,8]^, and Loey-Dietz syndromes^[9]^, and the associated pathological changes of the aortic tissue could be disruption of the medial elastic layers and marked medial fibrosis^[7,8]^. García-Macedo et al.^[6]^ reported a group of 27 patients with AAE and found that 23 (85.2%) were due to cystic medial necrosis, three (11.1%) were due to chronic aortic dissection, and one (3.7%) was due to syphilitic aortic aneurysm. Moreover, aortitis, including the giant cell aortitis (GCA), has been regarded a causative etiology of AAE and the resultant aortic regurgitation^[10]^. Annular progressive dilation as a consequence of AAE may also affect the long-term stability of a repair^[11]^. However, the roles that aortitis plays in the development of AAE remain uncertain, and the clinical features of AAE in patients with arteritis are unknown. This study aims to highlight the clinical features of AAE in patients with arteritis, the treatments of choice, and the causative relations between aortitis and AAE.

## METHODS

The Preferred Reporting Items for Systematic Reviews and Meta-analyses (PRISMA) statement guidelines were followed in this meta-analysis^[12]^. Publications were systematically searched in the PubMed, Highwire Press, and Cochrane Library databases from January 1990 to August 2018. The MeSH terms and keywords used to identify articles included “giant cell aortitis”, “Takayasu arteritis (TA)”, “Behçet disease”, and “annuloaortic ectasia.” Bibliographic references were tracked down to complete the literature retrieval. Thirty-three articles were found related to the topic and keywords in the literature search; and 28 articles, which met the inclusion and exclusion criteria during preliminary assessment, were included in the review. The exclusion criteria were: unrelative design to AAE (n=4) and no actual patient information available (n=1).

The data were carefully extracted for details of the study population, demographics, diagnostic techniques, clinical features, associated disorders, treatment, follow-up length, and patient’s prognosis. Quantitative data were presented as mean±standard deviation with range and median values. The intergroup differences were compared through the independent t-test, and the comparisons of frequencies were made by Fisher’s exact test. *P*<0.05 was considered statistically significant.

## RESULTS

In total, 28 articles^[7,13-39]^ including five case series and 23 case reports were collected, involving 57 patients. Forty-two (73.7%) patients from 23 articles^[7,13,14,17-26,28-38]^ had an AAE due to TA (two patients were associated with Marfan syndrome^[20,21]^ and one patient with ulcerative colitis^[38]^), 15 (26.3%) patients from five articles^[15,16,27,29,39]^ had an AAE due to GCA, but no article described an AAE due to Behçet disease. The patients’ gender was indicated for 48 patients: 11 (22.9%) were males and 37 (77.1%) were females (*χ*^2^=28.17, *P*<0.001), with a male-to-female ratio of 1:3^[36]^. The patients’ ages were 41.3±16.6 (range, 19-78; median, 38) years old (n=39). No age difference was found between male and female patients (42.5±17.9 years *vs*. 40.3±22.0 years, *P*=0.780). Patient demographics were listed in Table 1. A comparison between patients’ information from the five case series was shown in Table 2.

**Table 1:** Patients' demographics.

Variable	Result
Number of patients, n	57
Etiology, n (%)	
Takayasu arteritis	42 (73.7)
Giant cell aortitis	15 (26.3)
Patients' gender, male/female, n (%)	11 (22.9)/37 (77.1)
Patients' age, year, mean±standard deviation	41.3±16.6
Age of male patients, years	42.5±17.9
Age of female patients, years	40.3±22

**Table 2:** A comparison between the patients' information from the five case series.

**Author**	**n**	**Gender (male/female)**	**Age (year)**	**Aortitis' type (Takayasu arteritis/giant cell arteritis)**	**Symptom**	**Activity (n)**	**Aortic insufficiency (n)**	**Dimension of the sinus of Valsalva (mm)**	**Dimension of the aortic annulus (mm)**
Amano et al.^[14]^	8	1/7	39.4±3.6 (range, 26-57; median, 42)	8/0		2	8	53.4±4.3 (range, 35-70; median, 51)	24.9±0.5 (range, 23-27; median, 25)
Gelsomino et al.^[16]^	8			0/8	Circulatory or neurological symptoms (n=8)		8	Mean, 40	Mean, 26.8
Masaki et al.^[23]^	4	0/4	38-51	4/0		3	4		
Nakano et al.^[25]^	8	2/6	34.4	8/0		5			
Nesi et al.^[27]^	4	0/4	72±2.9 (range, 65-78; median, 72.5)	0/4	Dyspnea, atrial tachycardia (n=1), episode of heart failure (n=1), chest pain & dyspnea (n=1), respiratory distress (n=1)		4		
**Author**	**Dimension of the ascending aorta (mm)**	**Coronary artery lesion**	**Pathology of the aortic arch branch**	**Location of aortic aneurysm**	**Treatment**			**Outcome**	**Cause of death**
Amano et al.^[14]^	54.1±5.3 (range, 32-75; median, 50)			Root (n=8)	Bentall operation (n=8)			Died (n=1), recovered (n=7)	
Gelsomino et al.^[16]^	Mean, 52			Root+AA (n=8)	Bentall operation or AVR with AA replacement (n=8); steroid use for 1 year (n=2)			Died (n=1), recovered (n=9) out of 8 AAE patients & 2 non-AAE patients altogether	
Masaki et al.^[23]^			Obstructive lesions of the aortic arch branch (n=2)		Steroid & AVR (n=4)			Died (n=1), recovered (n=3)	Low cerebral perfusion (n=1)
Nakano et al.^[25]^	67.1	Coronary artery ectasia (n=2)	Stenosis and/or ectasia of the neck vessels (n=5)	AA (n=8)	Preoperative steroid therapy (n=2); Bentall operation (n=6), Bentall operation + CABG (n=1), Bentall operation + total arch replacement (n=1), postoperative steroid therapy (n=3)			Died (n=1), recovered (n=7)	Pulmonary failure (n=1)
Nesi et al.^[27]^				AA (n=1), AA + arch (n=2), AA + arch + DA (n=1)	Prolonged cortisone use & AA replacement + AVR (n=1), Bentall operation (n=3)			Died (n=1), recovered (n=3)	Multiple organ failure (n=1)

AA=ascending aorta; AAE=annuloaortic ectasia; AVR=aortic valve replacement; CABG=coronary artery bypass grafting; DA=descending aorta

Clinical symptoms were reported in 20 (35.1%) patients. In one article, symptoms of eight patients were described as circulatory or neurological^[16]^. In the remaining 12 patients, dyspnea and chest pains were common symptoms (Table 3). Twelve (20.1%) patients were in an active stage^[14,17,23,25,35]^.

**Table 3:** Nineteen symptoms presented by 12 patients.

Symptom	n (%)
Dyspnea^[17,27,29]^	5 (26.3)
Pain^[7,15,17,21,27]^ (chest pains in 4 patients^[7,17,21,27]^ and left popliteal pains in 1 patient^[15]^)	5 (26.3)
Heart failure^[20,27,38]^	3 (15.8)
Respiratory distress^[27]^	1 (5.3)
Palpitations^[17]^	1 (5.3)
Malaise^[17]^	1 (5.3)
Chest distress^[34]^	1 (5.3)
Cardiac tamponade^[7]^	1 (5.3)
Atrial tachycardia^[27]^	1 (5.3)

Aortic valve regurgitation was described in 44 (77.2%) patients. In 20 patients, the degree of aortic valve regurgitation was unspecified. In the remaining 24 patients: it was severe in 12 (50%)^[7,17,19,27,30,31,33,35,37]^, moderate or severe in eight (33.3%)^[16]^, moderate in two (8.3%)^[21,29]^, mild in one (4.2%)^[15]^, and trace in one (4.2%) patient^[18]^.

The morphology of aortic valve leaflets was mentioned in six (13.3%) patients: three were normal^[27]^ and three were abnormal (very thin^[19]^ in one, thinned out with lax cusps^[29]^ in one, and overstretch aortic valve leaflets in one patient^[37]^).

Dilation of the sinuses of Valsalva was noted in 20 (35.1%) patients^[7,14-17,20,37]^, with a dimension of 52.8±11.2 (range, 35-70; median, 50.5) mm (n=12).

The timing of occurrence of AAE was reported in only two (4.4%) patients, at two and five years after the diagnosis of GCA was made^[27]^. The dimensions of aortic annulus were described in 22 (38.6%) patients, measuring 31.5±12.2 (range, 23-60; median, 26) mm (n=15)^[7,14-19,34,37]^.

The morphology of the coronary artery was described in 13 (22.8%) patients: it was normal in seven (53.8%)^[7,26,27,29,37]^ and stenotic/occluded/ectatic in six (46.2%) patients^[18,19,21,25,32]^. Of the seven patients with normal coronary artery system, three had TA^[7,26,37]^ and four had GCA^[27,29]^, and all six patients with diseased coronary artery system had TA^[18,19,21,25,32]^.

The erythrocyte sedimentation rate was 40.5±31.8 (range, 12-70; median, 40) mm/hour (n=4)^[17,18,21,27]^, and the C-reactive protein was reported in 10 patients: it was normal in three^[15,18,22]^ and elevated in seven patients^[7,13,17,19,25,27,33]^. Among aortitis patients in active, active-to-inactive remission, and inactive phases, immunoglobulin G (IgG) and component 4 (C4) showed no significant intergroup difference. However, immunoglobulin A (IgA) and component 3 (C3) of patients in the inactive phase were the lowest among the three groups^[28]^.

Aortic arch branch lesions were present in 17 (29.8%) patients: 14 patients were with TA^[17-19,21,23-25,32,33]^ and three patients were with GCA^[27]^. Of the 14 TA patients, 13 (92.9%) patients had stenotic or occlusive lesions, one (7.1%) patient had both dilated and stenotic lesions^[17]^, and no patient had purely dilated lesions. Whereas, all three GCA patients had purely dilated lesions of the aortic arch branches (*χ* ^2^=18, *P*<0.001).

Seven (12.3%) patients had no aortic aneurysms^[15,19,23,28]^, with one of them having a left popliteal artery aneurysm instead that required surgical aneurysmectomy^[15]^. Aortic aneurysms were found in the remaining 50 (87.7%) patients, with the ascending aorta aneurysm being the most common (Table 4).

**Table 4:** Aortic aneurysm in 48 patients with annuloaortic ectasia.

Aortic aneurysm	n (%)
AA^[18,20,21,25,27,29,34,37,38]^ (1 patient had mixed stenosis and dilation of the aorta^[34]^)	16 (33.3)
Root^[7,13,14,22,31-33]^	12 (25.0)
Root, AA^[16]^	8 (16.7)
AA, arch^[26,27]^	3 (6.3)
AA-arch-DA^[17,24,27]^	3 (6.3)
Arch^[13]^	1 (2.1)
Root-arch^[35]^	1 (2.1)
Root, arch^[30]^	1 (2.1)
Root, AA, arch, DA^[37]^	1 (2.1)
Root, AA, arch^[39]^	1 (2.1)
Entire aorta^[22]^	1 (2.1)

AA=ascending aorta; DA=descending aorta

One patient’s treatment was not mentioned^[18]^, one patient was treated conservatively with nonsteroidal medications and died suddenly due to rupture of aneurysm of the sinuses of Valsalva^[10]^, and one patient had popliteal arterial aneurysmectomy, in whom AAE was not exacerbated at 18-month follow-up^[15]^. The remaining 54 (94.7%) patients underwent cardiac operations (one of them received a hybrid procedure^[39]^), and the most common surgical procedure performed was the Bentall operation (Table 5).

**Table 5:** Cardiac surgical procedures performed in 54 patients with annuloaortic ectasia.

Cardiac operation	n (%)
Bentall^[13,14,22,25-27,29,31,33,34,38]^(staged thoracoabdominal aorta replacement in 1 patient^[22]^)	25 (46.3)
Bentall/aortic valve replacement + AA replacement^[16]^	8 (14.8)
Bentall + arch replacement^[13,25,30,35,39]^ (with emergency endovascular repair of DA in 1 patient^[39]^)	6 (11.1)
AVR^[23]^	4 (7.4)
Bentall + CABG^[19,21,25,32]^	4 (7.4)
AVR + AA replacement^[27,36]^ (with endarterectomy & patch plasty to the innominate artery in 1 patient^[36]^)	2 (3.7)
Cabrol operation^[20,37]^	2 (3.7)
Bentall + arch replacement + arch branch replacement^[17]^	1 (1.9)
Bentall + arch + proximal DA replacement^[24]^	1 (1.9)
Unspecified^[28]^	1 (1.9)

AA=ascending aorta; AVR=aortic valve replacement; CABG=coronary artery bypass grafting; DA=descending aorta

The outcomes were reported for 53 (93.0%) patients: four (7.5%) were complicated (with anastomotic dehiscence) at 7.1±8.8 (range, 0.5-20; median, 4) years (n=4) after the operation^[26,31,32,35]^, six (11.3%) patients died^[7,14,23,25,27,39]^, one (1.9%) patient remained unchanged^[15]^, and 42 (79.2%) patients recovered^[13,14,16,17,19,22-25,27,29,33,34,36-38]^. No significance was found in the surgical outcomes between patients with TA and those with GCA (Table 6).

**Table 6:** A comparison of surgical prognoses between patients with Takayasu arteritis and those with giant cell arteritis.

Primary disease	Recovered	Complicated	Died
Takayasu arteritis	31 (79.5)	4 (10.3)	4 (10.3)
Giant cell arteritis	12 (85.7)	0 (0)	2 (14.3)
Χ^2^	0.26	1.55	0.17
*P* value	0.609	0.213	0.683

*Fisher's exact test.

In TA patients, extensive intimal fibrosis, loss or fragmentation of the elastic lamina, and patchy infiltration of inflammatory cells were found in the aortic tissue^[7,17,19]^, and the aortic valve leaflet showed mucoid degeneration with inflammatory cell infiltration^[37]^. In the GCA patients, cystic medial degeneration and dystrophic calcification^[21]^, and plasma cells and lymphocytes with central hyalinized connective tissues^[29]^ of the aortic wall were noted.

## DISCUSSION

It has been reported that rolling of free cusps of the aortic valve was a unique finding of AAE^[34]^. The aortic valve can also be thin, and the aortic valve ring can expand to 60 mm in diameter^[19]^. At the histologic examination, the aortic leaflets showed signs of inflammation with mononuclear cellular infiltrate, even though true granulomatous-like inflammatory reaction with multinucleated giant cells was not seen.

Dilation of a coronary artery may be secondary to AAE or share a similar etiology to AAE. As a result of cystic medial necrosis of the aorta, AAE is the most common cardiovascular manifestation of Marfan syndrome^[40]^. Becker and van Mantgem^[41]^ studied the morphology of coronary arteries of Marfan patients and found out that the pathological changes of the coronary artery were cystic medial necroses, similar to those seen in the aorta of arteritis patients. However, in patients without Marfan stigmata, coronary artery ectasia may also be present^[42]^.

The Bentall procedure is often indicated for aortic regurgitation associated with AAE^[43]^. The David operation with native aortic valve preservation is useful for preventing some complications associated with artificial heart valves^[44]^. Thus, a “valve-sparing” operation could be used for such patients. However, these patients were young, and the dystrophic changes of the cusps were mild; furthermore, a David operation should be the treatment of choice whenever possible.

Composite graft anastomosed to the aortic annulus with buttress sutures reinforced with Dacron felt may potentially decrease the risk of prosthetic detachment^[33]^, i.e., dehiscence of the composite graft from the aortic annulus^[35]^. Long-term results for aortic valvuloplasty or redo-operation after aortic valvuloplasty in TA patients even in the active phase can be good^[17]^. At the site of the external reinforcement of the aneurysm, further expansion of the ascending aorta may be inavoidable, and an aortic aneurysm may develop at the interface of the aorta and Dacron fabric for wrapping. These complications can be difficult to manage when external reinforcement and aortic valvuloplasty are undertaken during active inflammatory AAE^[17]^.

Postoperatively, anastomotic dehiscence may occur at the distal anastomosis of Dacron graft and at the non-coronary cusp site of the proximal composite graft. Paravalvular leaks, another surgical complication, have been considered to have begun five years after the initial operation with inflammatory deterioration^[31]^. Clinical observations revealed that previous saphenous vein and aortoaxillary bypass grafts were markedly enlarged, the prosthetic valve was detached from the annular ring, and the motion was restricted by small thrombus and the remnant of the right coronary cusp^[26]^. In the patient cohort of Evans et al.^[10]^, the aortic valve cusps were macroscopically normal and valve incompetence was presumably caused by the lack of leaflets coaptation secondary to aneurysmal dilation of the aortic root. Late death (50%) occurred at a late stage up to a mean of 23.8 months due to severe complications, such as valve detachment or postoperative pseudoaneurysm. Comparisons of preoperative immunological values between the survival and late death groups were made, and the immune biomarkers, such as IgG, IgA, C3, and C4, were significantly higher in the late death group. It was explained that the insertion of the rigid prosthesis in the fragile position might stimulate immunological reactions due to latent inflammation^[28]^.

Shiono et al.^[31]^ reported that, in initial Bentall procedures, both coronary ostia were directly anastomosed to composite valved graft and wrapped with native aortic wall; the coronary button was lacerated in five years. To avoid laceration of coronary anastomosis, Carrel’s button technique is advocated in redo-operations^[30]^. Additionally, the composite graft anastomosed to the aortic annulus with buttress sutures reinforced with a Dacron felt was also recommended. Both coronary orifices were able to be reconstructed with small-sized Dacron grafts, interposed from the coronary orifices to the composite graft^[33]^.

This study provides typical clinical features of AAE in arteritis. The morphology of the aortic valve leaflets was normal in half of the patients, and the valves were thin and overstretched in the other half. Most patients had an aortic aneurysm. Half of the patients had severe aortic regurgitation, and one-quarter of them had dilation of the sinuses of Valsalva. It could be deduced that the inflammatory cellular infiltrate and enhanced immune biomarkers became the intriguing contributions of aortitis to the pertinent pathological changes of AAE. TA was prone to develop coronary artery lesions, while GCA was not. The aortic branch lesions in TA were mostly stenotic or occlusive, while in GCA, they were all dilated lesions. Kermani et al.^[45]^ also reported the same findings of predominant stenotic/occlusive lesions in TA, whereas more common aneurysmal disease was noted in GCA. Nevertheless, the pertinent mechanisms leading to the disparity of the vascular changes between the two lesions were not interpreted.

The shortage of information about AAE in arteritis patients in the literature constitutes the major drawback of this study. Currently, some important aspects of AAE in arteritis have not been satisfactorily answered. These questions include the timing of AAE development after arteritis onset, the geometric morphometrics of the aortic valve annulus, the hemodynamic impact of the geometric morphometrics, the pathology of the aortic valve leaflets, the durability of aortic valve repair in relation to these morphometric and pathological features, more feasible measures for the prevention of postoperative anastomotic dehiscence, and comparisons with AAE of other etiologies. These questions are anticipated to be answered in the future.

## CONCLUSION

Arteritis can cause AAE, which is characterized by dilated aortic valve annulus, sinuses of Valsalva, and ascending aorta. Some patients show coronary artery and aortic arch branch lesions. The inflammatory cellular infiltrate and enhanced immune biomarkers can be the intriguing contributions of aortitis to the pertinent pathological changes of AAE. The mechanisms of the predominant stenotic/occlusive lesions in TA and more common aneurysmal disease in GCA remain uncertain. Most patients (94.7%) require surgical treatment with steroid therapy. However, long-term follow-up results show a higher anastomotic dehiscence rate, particularly in patients with TA. Further morphometric and pathological research on AAE in arteritis should be undertaken.

**Table:** 

Author's roles & responsibilities	
SMY	Conception or design of the work; acquisition, analysis, or interpretation of data for the work; drafting the work or revising it critically for important intellectual content; agreement to be accountable for all aspects of the work in ensuring that questions related to the accuracy or integrity of any part of the work are appropriately investigated and resolved; final approval of the version to be published
HL	Interpretation of data for the work; final approval of the version to be published
